# Calendar

**DOI:** 10.1155/S0962935195000500

**Published:** 1995-07


Mediators of Inflammation 4, 298–305 (1995)

### IL-4 induces cAMP and cGMP in human monocytic cells

B. Dugas,[1] N. Paul-Eugene,[2] K. Yamaoka,[4] C. Amirand,[3] C. Damais[2] and J. P. Kolb[4,CA]

[1]URA CNRS 625, Hôpital Pitié-Salpêtrière, 75013 Paris; [2]INSERM U313, Centre BioMédical des Cordeliers, 75006 Paris; [3]URA CNRS 198, and [4]INSERM U365, Institut Curie, 75005 Paris, France

[CA]Corresponding Author

**HUMAN monocytes, preincubated with IFN-γ, respond to IL-4 by a cGMP increase through activation of an inducible NO synthase. Here, IL-4 was found to induce an accumulation of cGMP (1 – 3 min) and cAMP (20 – 25 min) in unstimulated monocytes. This was impaired with NOS inhibitors, but also with EGTA and calcium/calmodulin inhibitors. These results suggest that: (1) IL-4 may stimulate different NOS isoforms in resting and IFN-γ activated monocytes, and (2) cAMP accumulation may be partially dependent on the NO pathway. By RT-PCR, a type III constitutive NOS mRNA was detected in U937 monocytic cells. IL-4 also increased the $[Ca^{2+}]_i$ in these cells. Different NOS may thus be expressed in monocytic cells depending on their differentiation and the signals they receive.**

**Key words:** $[Ca^{2+}]_i$, cAMP, cGMP, Human monocytes, IL-4, NO synthase.

### Introduction

Interleukin-4 (IL-4) is a 20 kDa glycoprotein, produced by activated T lymphocytes ($Th_2$), mast cells, basophils and other cell types, which displays a broad spectrum of biological activity on haematopoietic cells.[1] On monocytes/macrophages, IL-4 enhances the expression of MHC class II antigens, adhesion molecules of the LFA-1 (leucocyte functional antigen) family[2] and CD23.[3] In contrast, IL-4 down-regulates the expression of FcγRI (CD64) and FcγRII (CD32) and inhibits the induction of FcγRIII (CD16) by IFN-γ[4] or TGF-β.[5] IL-4 also down-regulates the expression of CD14 on monocytes.[6] IL-4 acts as an anti-inflammatory mediator by decreasing the release of IL-1, TNF-α, IL-6, IL-8 and prostaglandin $E_2$ by human monocytes and by reducing their capacity to produce reactive oxygen species.[7–12] IL-4 also induces the secretion of G- and M-CSF by monocytes[13] and triggers their differentiation into macrophage-like cells type *in vitro*.

The binding component of the IL-4 receptor belongs to the family of haemapoietin receptors[14,15] and is associated, at least in some cell types, to the γ chain previously reported for the IL-2 receptor.[16–22] The importance of protein tyrosine phosphorylation in IL-4 signalling is now well recognized[23–28] and recently, the Jak-3[29] and Lsk[30] have been identified as the major tyrosine kinases involved. Several transcription factors are selectively activated through IL-4-induced tyrosine phosphorylation.[31,32]

We recently described that IL-4 induced an accumulation of cGMP in monocytes of certain donors, which was greatly enhanced by preincubation with IFN-γ and was dependent on the L-arginine-dependent pathway of nitric oxide synthase (NOS) activation, since it was markedly reduced in the presence of NOS inhibitors.[33] We also reported that IL-4 displayed a biphasic effect on the capacity of cultured monocytes to release nitrite in their supernatants.[34] In the present work, we have further analysed the effect of IL-4 on the production by monocytic cells of the cyclic nucleotides cAMP and cGMP.

### Materials and Methods

*Reagents:* Recombinant human IL-4 obtained from *Escherichia coli* (specific activity, $1 \times 10^7$ U/mg, 1 U = 1 ng) was purchased from Immugenex (Los Angeles, CA, USA). Isobutyl methyl xanthine (IBMX), EGTA (ethylene glycol-*bis*(β-aminoethylether) N,N,N′,N′-tetraacetic acid) and the two inhibitors of calmodulin, calmidazolium and W-7, were purchased from Sigma (Saint Louis, MO, USA). $N^G$-mono-methyl-L-arginine (L-NMMA) and $N^G$-nitro-L-arginine (L-NA), two *N*-guanido-substituted analogues of L-arginine, competitive inhibitors of NO synthase, were purchased from Calbiochem and Sigma, respectively. The neutralizing anti-IL-4 mAb was from Immunotech (Luminy, France).

*Isolation of human monocytes:* Blood samples were obtained from cytapheresis of normal donors, as residues of platelet preparations, thorugh the courtesy of the blood transfusion centres of Créteil and Hôtel-Dieu. Blood donors

© 1995 Rapid Science Publishers did not show contamination by HIV and hepatitis B, or suffer from manifestations of allergy. Otherwise their clinical and immunological status was not known. Peripheral blood mononuclear cells (PBMC) were purified as previously described.[23] Briefly, blood samples were centrifuged at 200 × ***g*** for 10 min in order to eliminate the residual contaminating platelets. Then, samples were diluted in RPMI 1640 medium (Bioproduct, Les Ulis, France) and PBMC were harvested after a 20 min centrifugation at 800 × ***g*** on Ficoll–Hypaque (Pharmacia, Uppsala, Sweden) gradient. Adherent cell populations were obtained by incubation of PBMC ($1 \times 10^7$ cells/ml) in Petri dishes (Nunc, Rocksille, Denmark) for 1 h at 37°C in RPMI 1640 medium supplemented with 10% FCS (Gibco, Paisley, UK). Monocytes were harvested by scraping the plates with a rubber policeman after addition of cold phosphate-buffered saline solution (PBS) containing 1 mM EDTA. Cell preparations were composed of more than 95% viable monocytes as assessed by the Trypan blue exclusion method and nonspecific esterase staining. Other contaminating cells were lymphocytes and granulocytes which never exceeded 5% of the total cell number, whereas platelet contamination was undetectable.

*Monocytic cell line:* U937 promonocytic cells were maintained in RPMI-1640 medium (Gibco, Paisley, Scotland) supplemented with 5% FCS (Gibco: LPS levels <0.2 ng/ml), 2 mM L-glutamine, 1 mM pyruvate, penicillin 100 U/ml, streptomycin 100 μg/ml (Flow Laboratories, Rockville, MD) in a 5% $CO_2$-humidified atmosphere at 37°C. For experiments, the cells were collected, washed and counted with a Coulter counter ZM, equipped with a Coultronic 256 channelizer (Coultronics, Margency, France).

*Determination of cAMP and cGMP:* For measuring the cytoplasmic cGMP and cAMP levels in whole cells, monocytes were resuspended at $7 \times 10^6$/ml in RPMI 1640 medium supplemented with 200 μM of the phosphodiesterase inhibitor IBMX and incubated at 37°C for 15 min. After the incubation period, $1 \times 10^6$ cells (135 μl) were distributed in duplicate or triplicate samples in Eppendorf microtubes. Fifteen μl of the different ligands were added for various periods of incubation at 37°C in a water bath. The reactions were stopped by addition of 25 μl of 35% perchloric acid (PCA) and immediate cooling on ice. The tubes were allowed to stand at 0°C for 15 min, then spun at 3 000 × ***g*** for 15 min. The pellets were discarded and the supernatants neutralized with KOH. The $KClO_4$ precipitates were removed by a second centrifugation at 3 000 × ***g*** for 15 min at 4°C. The neutralized supernatants were kept frozen at −70°C until cGMP and cAMP determinations. After acetylation of the samples, the concentrations of cGMP and cAMP were determined by specific radioimmunoassays, according to the specifications of the manufacturers (kits from Amersham France, Les Ulis or from NEN, Dupont, Dreieich, Germany). The significance of the results was analysed by the Student's *t*-test, adapted for small samples.

*Identification of constitutive NO synthase in monocytic cells by reverse transcription-polymerase chain reaction:* Total RNA samples were extracted from monocytes and U937 cells by a modification of the technique of MacDonald *et al.*[35] Briefly, cells were washed twice in ice-cold PBS then resuspended in 1.5 ml of 5 M guanidinium thiocyanate (Fluka, Buchs, Switzerland). After sonication for 20 s in a Vibra Cell sonifier (Bioblock Scientific, Illkirch, France) at 40% maximal power, the cell lysates were layered on 1.5 ml cushions of 5.7 M CsCl dispensed in polycarbonate tubes and centrifuged at 20 000 × ***g*** for 3 h at 20°C in the TLA 100.3 rotor of a TL 100 ultracentrifuge (Beckman Instruments). After two washings with formamide/ethanol (30/70 v/v), the pellet was dried and resuspended in 200 μl of TE buffer (Tris 10 mM, EDTA 1 mM). After addition of 0.1 vol of 3 M sodium acetate, the samples were mixed with 2 vol of absolute ethanol and then placed at −70°C overnight to precipitate total RNA. Sedimentation at 13 200 × ***g*** for 30 min at 4°C was performed and the resulting total RNA pellet was dried, resuspended in diethyl pyrocarbonate (DEPC)-treated deionized water and its concentration was estimated by spectrophotometry. Total RNA (1 – 2 μg) from monocytes and U937 cells was reverse transcribed using random hexanucleotides as primers (First-strand cDNA synthesis kit, Amersham France, Les Ulis, France). The cDNA generated was then amplified by the polymerase chain reaction. After an initial denaturation step at 94°C, 30 PCR cycles were performed as follows: 25 s at 95°C, 1 min at 57°C, 1 min at 72°C, followed by a final extension step of 5 min at 72°C. The following oligonucleotide primers, based on the human endothelial cells cNOS cDNA sequence, were used to amplify the monocytes and U937 NOS cDNA: forward, 5′-GCA TCA CCT ATG ACA CCC TCA GCG-3′; reverse, 5′-AGC TCG CTC TCC CTA AGC TGG TAG G-3′. These primers define a fragment of 277 bp. Oligonucleotides were synthesized by Genset (Paris, France), except for control primers for human β-actin which were obtained from Stratagene (La Jolla, CA, USA). Products of the reverse transcriptase-polymerase chain reaction (RT-PCR) were analysed by agarose (1.4%) gel electrophoresis in the presence of BET.

*Determination of $[Ca^{2+}]_i$:* Cells were loaded with 10 μM of the fluorescent probe Fluo 3-acetoxymethylester (Fluo 3-AM) for 1 h at 37°C; the cells were washed and resuspended in RPMI-1640 medium, without phenol red, containing 10 mM HEPES. Fluorescence intensity (in arbitrary units) of Fluo-3 AM-loaded U937 cells was recorded as a function of the wavelength between 500 and 600 nm (maximum emission of Fluo-3/Ca complex: 535 nm), as described previously.[36] The free calcium concentration $[Ca^{2+}]_i$ in the cells is related to the fluorescence intensity ($F$) by the following equation:

$$[Ca^{2+}]_i = K_d \times (F - F_{min})/(F_{max} - F)$$

where $F_{max}$ is determined by measuring fluorescence after the cells are lysed in 0.1% Triton X-100 (Sigma), whereas $F_{min}$ is determined after addition of 10 mM EGTA. The effective constant $K_d$ of Fluo-3 in solution has been reported to be 400 nM. Fluo-3 is analysed in the visible range, thus the problems associated with the use of UV light are avoided. In addition, the large increase of the fluorescence emission by binding of $Ca^{2+}$ to Fluo-3 allows a great sensitivity for measuring small variations in calcium concentration. However, upon binding of $Ca^{2+}$, there are no shifts in its excitation or emission spectrum, which makes Fluo-3 non-ratioable and therefore will compromise the accuracy of absolute $[Ca^{2+}]_i$ determinations. For these reasons, our data have been presented as fluorescence intensities.

### Results

*IL-4 stimulates a biphasic cyclic AMP accumulation in human monocytes:* Incubation of freshly isolated monocytes with IL-4 was found to result in a delayed accumulation of cAMP, starting at 10 – 15 min and peaking about 20 – 25 min after addition of the cytokine. For some donors, a transient peak of cAMP was also observed after 1 min of stimulation, as shown in the typical response presented in Fig. 1. After a phase of plateau until 30 min, a progressive decrease occurred and a return to the basal value was achieved around 1 h after the beginning of the stimulation (not shown). The increase in cAMP content over background was usually in the range 50 – 300% ($n = 11$). This late increase in cAMP was dependent on the concentration of IL-4, as presented in Fig. 2. When the experiments were performed in the presence of 10 μM indomethacin, a cyclooxygenase inhibitor, the IL-4-induced cAMP response was not significantly affected, indicating that the increase in cAMP was not secondary to a release of prostaglandins (not shown).

*IL-4 stimulates cyclic GMP accumulation in human monocytes:* The kinetics of IL-4 driven cAMP and cGMP accumulation were compared in unstimulated monocyte preparations from the same donors and typical results are presented in Fig. 3. It was regularly observed that the peak of cGMP accumulation (between 3 and 10 min) preceded that of late cAMP accumulation. For eleven donors tested, the range of cGMP accu-

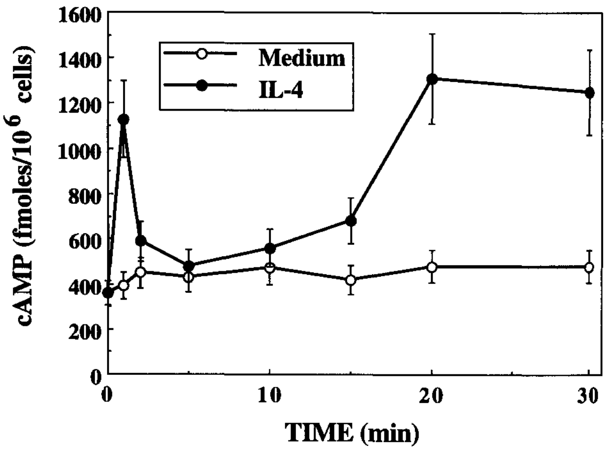

FIG. 1. Kinetics of IL-4-induced cAMP accumulation in normal human monocytes. Monocytes were incubated in triplicate samples without or with IL-4 (10 ng/ml) for the times indicated, and cAMP determination was performed as described in Materials and Methods. Results are from one representative experiment out of eleven, except for the detection of the first cAMP transient which was observed only for three out of five donors where this early time point was tested.

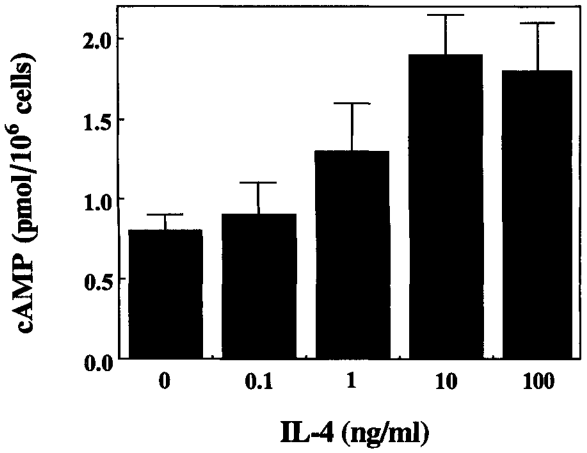

FIG. 2. Dose-dependent induction of cAMP in monocytes by IL-4. Human monocytes were stimulated for 20 min with medium alone or with varying concentrations of IL-4 (0.1 – 100 ng/ml) and cAMP accumulation was determined as previously described. Results are the mean ± S.D. of an experiment in triplicate samples, representative of three.

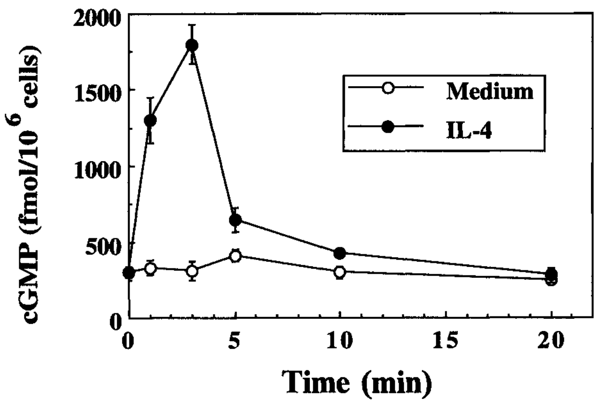

FIG. 3. Kinetics of IL-4-driven cGMP accumulation in human monocytes. Monocytes were stimulated with IL-4 (10 ng/ml) for the times indicated and cGMP concentrations were determined by RIA on perchloric extracts, as described by Materials and Methods. Data are mean ± S.E.M. of triplicate samples from one experiment representative of nine where a significant cGMP response could be detected.

mulation, expressed as a percentage of the control response in the presence of medium alone, was 132 – 396% (mean ± S.D. = 236 ± 80; median = 225). The induction of cAMP and cGMP by IL-4 was specific, since it was abrogated in the presence of 50 μg/ml of a neutralizing anti-IL-4 antibody, but not of an isotype-matched irrelevant antibody as shown in Table 1.

*Inhibitors of the nitric oxide synthase pathway impair the IL-4-driven cyclic nucleotides accumulation in resting monocytes:* The heterodimeric soluble guanylyl cyclases, which convert GTP into cGMP, are activated by NO-generating compounds through interactions of the NO radical with their haeminic prosthetic groups. In order to test whether the observed accumulation of cGMP in response to IL-4 was due to a generation of NO, two classical inhibitors of the L-arginine pathway of NO synthase activation were added to unstimulated monocytes prior to their incubation with IL-4: L-NMMA, and nitro-L-arginine which, at variance with L-NMMA, does not compete with L-arginine for its transport into the cells. As seen in Table 2, the IL-4-driven cGMP accumulation was almost totally abolished in the

Table 1. IL-4-driven cyclic nucleotide accumulation in human monocytes is inhibited with a specific neutralizing anti-IL-4 antibody

| | cAMP (fmol/10$^6$ cells) | cGMP (fmol/10$^6$ cells) |
|---|---|---|
| Medium | 460 ± 50 | 410 ± 110 |
| IL-4 | 1500 ± 190 | 1220 ± 130 |
| IL-4 + anti-IL-4 | 510 ± 70 | 510 ± 80 |

Monocytes were incubated for 3 min (cGMP) or 20 min (cAMP) with medium, IL-4 (10 ng/ml), or IL-4 and anti-IL-4 mAb (20 μg/ml). The reactions were then stopped and cells extracts were determined for their content in cAMP and cGMP as described in Materials and Methods. Results are the mean ± S.E.M. of triplicate samples from one representative experiment out of two.

presence of these competitive inhibitors of L-arginine for the NOS. Interestingly, IL-4-driven cAMP accumulation was also markedly affected with L-NMMA as shown in Fig. 4, suggesting that both cAMP and cGMP production were related to the NO pathway.

*Effect of calcium chelator and calmodulin inhibitors on IL-4 driven cGMP accumulation:* Several NOS isoforms have been described, which differ in their sensitivity to calcium for the stimulation of their catalytic activity. Various inhibitors of the Ca/CaM complex were thus tested

Table 2. Suppression of IL-4-driven cGMP accumulation by nitric oxide synthase inhibitors

| | Expt 1 | Expt 2 | Expt 3 | Expt 4 | Expt 5 | Expt 6 |
|---|---|---|---|---|---|---|
| Medium | 410 | 390 | 270 | 830 | 930 | 3500 |
| IL-4 | 1900 | 900 | 730 | 2930 | 3730 | 5530 |
| IL-4 + L-NMMA | NT | 340 | 300 | 800 | 700 | 3140 |
| Inhibition (%) | - | 100 | 92 | 100 | 100 | 100 |
| IL-4 + L-NA | 900 | 350 | 285 | NT | 690 | NT |
| Inhibition (%) | 67 | 100 | 96 | - | 100 | - |

Monocytes from six different donors were incubated for various times with medium, IL-4 (10 ng/ml), or IL-4 and either 1 mM of $N^G$-monomethyl-L-arginine (L-NMMA) or $N^G$-nitro-L-arginine (L-NA). The reactions were then stopped and cell extracts were determined for their content in cGMP as described in Materials and Methods. Results are the mean of triplicate samples and are expressed as fmoles cGMP/10$^6$ cells, at the peak of the response (1 – 2 min).

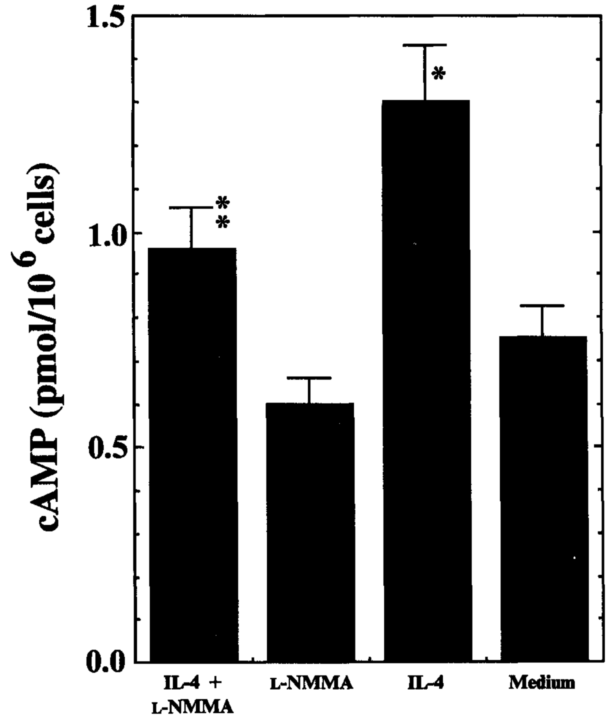

FIG. 4. Effect of L-NMMA on IL-4-induced cAMP accumulation in human monocytes. Monocytes were preincubated or not with L-NMMA (1 mM) for 30 min at 37°C, then stimulated for 20 min with medium or IL-4 (10 ng/ml). Results are mean ± S.E.M. of triplicate samples from one representative experiment out of seven. *$p < 0.01$; **$p < 0.05$.

and, as seen in Table 3, the IL-4-stimulated cGMP increase was greatly reduced either in the presence of the calcium chelator EGTA or of the calmodulin inhibitor W7, both reagents being ineffective alone at the concentrations tested. Similar results were obtained with calmidazolium, another inhibitor of the Ca/CaM complex (not shown). These results therefore suggested that the NOS involved may be different from the inducible type II NOS detected in IFN-γ-preincubated monocytes[33] and could be activated through variations in intracytoplasmic calcium concentration and therefore be related to the constitutive NOS isoforms.

*IL-4 stimulates an increase in intracytoplasmic free calcium $[Ca^{2+}]_i$ in unstimulated monocytic cells:* Inasmuch as cNOS are regulated via transient calcium increases, we measured the effects of IL-4 on the $[Ca^{2+}]_i$ in a monocytic cell line, U937 cells, which behave like blood monocytes with regard to cyclic nucleotide accumulation in response to IL-4. As seen in Fig. 5, addition of IL-4 to Fluo-3-loaded resting U937 cells resulted within 20 s in a marked increase in $[Ca^{2+}]_i$. Although Fluo-3 is not ratioable, an approximate calculation gives a value of 78 nM for the $[Ca^{2+}]_i$ in the resting state which increased to about 1070 nM after IL-4 administration. These results were confirmed by micro-spectrofluorometric techniques at the single cell level; high increases in fluorescence intensity were recorded following IL-4 addition to U937 cells, although we observed that the response of individual cells was heterogeneous, some cells being unresponsive to IL-4 stimulation (data not shown). These results demonstrate that IL-4 is able to elicit an increase in $[Ca^{2+}]_i$ in monocytic cells, a prerequisite for the activation of cNOS in these cells. Indeed, the catalytic activity of the constitutive type I and III cNOS are regulated by the Ca/CaM complex, through an increase in the intracellular concentration of free calcium $[Ca^{2+}]_i$ evoked by specific hormones or neurotransmitters. When calcium ionophores such as ionomycine and A23187 were added to monocytes, however, no increase in cGMP over the basal concentration was detected, nor any significant release of nitrite in the supernatant of these cells; the same negative results were obtained when monocytes or U937 cells were incubated in the presence of thapsigargin, an inhibitor of the endoplasmic reticulum calcium ATPase, which constitutes another way to raise the intracytoplasmic $[Ca^{2+}]_i$. An increase in $[Ca^{2+}]_i$ is therefore not sufficient, alone, to trigger cGMP accumulation by monocytic cells.

Table 3. Suppression of IL-4-driven cGMP accumulation in human monocytes by calcium chelator and inhibitor of the Ca/CaM complex

| | Expt 1 | Expt 2 | Expt 3 | Expt 4 | Expt 5 | Expt 6 |
|---|---|---|---|---|---|---|
| Medium | 650 | 1000 | 670 | 530 | 1000 | 3500 |
| IL-4 | 6160 | 2670 | 1160 | 1280 | 5670 | 5400 |
| IL-4 + W7 | 670 | 1070 | 800 | 715 | 1690 | 2810 |
| IL-4 + EGTA | 630 | 930 | 710 | NT | 1340 | NT |

Monocytes from six different donors were stimulated for various times with medium, IL-4 (100 ng/ml), IL-4 (100 ng/ml) and W-7 (10 μg/ml), or IL-4 (100 ng/ml) + EGTA (2 mM). Reactions were stopped and cell extracts were analysed for cGMP content as described in Materials and Methods. Results are mean of triplicate samples at the peak of the response (1 – 2 min) and are expressed as fmoles/$10^6$ cells. NT: not tested.

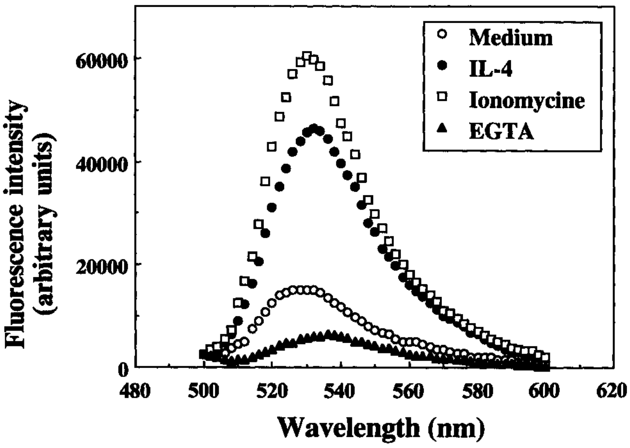

FIG. 5. IL-4 stimulates an increase in free calcium concentration in U937 cells. Fluorescence intensity (in arbitrary units) of Fluo-3 AM-loaded U937 cells was recorded as a function of the wavelength. U937 cells were successively exposed to medium, IL-4 (50 ng/ml), ionomycine (10 μM), and finally EGTA (10 mM). Data are from one representative experiment out of three.

*Detection by RT-PCR of type III cNOS mRNA in human monocytic cells:* Total RNA from human monocytes and U937 cells were reverse transcribed and amplified by the polymerase chain reaction, using primers specific for the constitutive endothelial human NO synthase. As seen in Fig. 6, a fragment of 277 bp was readily detected in U937 cells, which corresponds to the size expected from the sequence of the endothelial NO synthase. Although much weaker than that of U937 cells, this band was also observed with monocytes (not shown). Treatment of U937 cells with either IL-4, or IL-4 and anti-C23 mAb, or IFN-γ, did not modify the expression of cNOS.

### Discussion

We have previously observed that, for a fraction of the donors tested, IL-4 induced a cGMP increase in unstimulated monocytes; a much higher response was detected when monocytes were preincubated with IFN-γ; this was inhibited in the presence of L-NMMA and was not affected by calcium chelation, suggesting the activation of

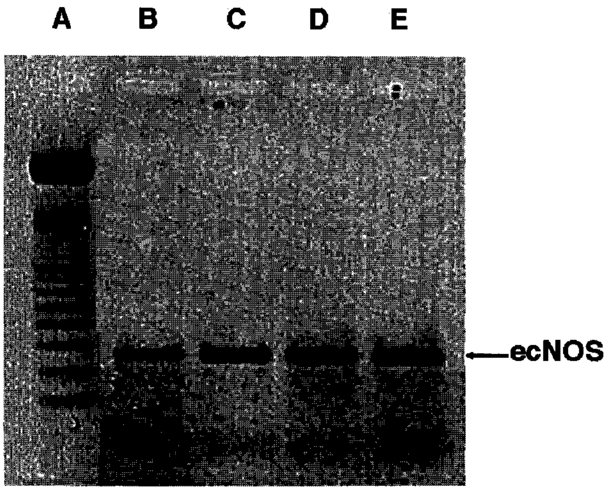

FIG. 6. Detection by RT-PCR of a type III (endothelial) constitutive cNOS mRNA in human monocytic cells. Total RNA (1 – 2 μg) from monocytes and U937 cells was reverse transcribed and amplified by PCR using specific primers for human ecNOS. RT-PCR products were analysed by agarose (1.4%) gel electrophoresis in the presence of BET. Lane A: size markers (100 bp ladder). Lane B: unstimulated U937 cells. Lane C: IL-4-activated U937 cells. Lane D: IL-4 and anti-CD23 mAb-activated U937 cells. Lane E: IFN-γ-activated U937 cells.

an iNOS.[33] In addition, IL-4 induced a slight production of nitrite by monocytes, which was potentiated by IFN-γ and inhibited by L-NMMA. These results suggested that the sequential exposure of monocytes to IFN-γ and IL-4 elicited the release of NO from L-arginine, which in turn was able to stimulate soluble guanylate cyclase.[33] We have also reported that IL-4 induced an heterogeneous and late accumulation of nitrite in monocyte supernatants.[34] In the present work, we show that IL-4 induces cAMP and cGMP generation in unstimulated human monocytes. A rather large heterogeneity in the response of the different individuals was observed, which may be explained by the fact that, except for the absence of HIV or hepatitis infection and of allergy, the immunological status of these donors was largely unknown. One cannot exclude the possibility that some of these volunteers were primed otherwise which may explain the variation in the response observed and the biphasic cAMP response which was detected for some of them. We have no direct explanation for this early cAMP increase; tentatively, one may speculate that it could be achieved through a cross stimulation of $\beta_2$ receptors, inasmuch as we have previously demonstrated a functional interplay between the response of monocytes to IL-4 and to $\beta_2$ agonists. Hart *et al.*[37] did not detect significant cAMP accumulation in elutriation-enriched monocytes stimulated by IL-4 or IL-4 plus LPS; however, their experiments were performed with low concentration of IL-4 (1 ng/ml), that, in our hands too, only triggered a slight cAMP increase, whereas higher IL-4 concentrations stimulated significant increases in both cyclic nucleotides.

The IL-4 receptor binding unit and its γ chain-associated component do not belong to the family of receptors with seven spanning domains, capable of interacting with Gsα and leading to the activation of adenylate cyclase (AC). Furthermore, whether the associated γ chain has been detected in lymphoid cells, it appears to be absent in monocytic cells, as revealed by RT-PCR.[38,39] The late accumulation of cAMP suggested therefore an indirect mechanism of action of IL-4. Activation of AC via the induction of prostaglandin by IL-4 was ruled out, since the IL-4-driven cAMP accumulation was not significantly affected in the presence of indomethacin, an inhibitor of cyclooxygenase. Alternatively, the observed IL-4-induced cAMP accumulation could result from AC stimulation through the activation of PKC or of the calcium/calmodulin complex.[40]

The role of protein kinase C (PKC) and cyclic nucleotides in IL-4 signalling is controversial. Arruda and Ho[41] presented evidence that, in human monocytes, IL-4 signalling involved PKC translocation to a nuclear fraction, without parallel increase in $[Ca^{2+}]_i$, suggesting a possible role for phosphatidylcholine hydrolysis in this process; the same group has shown recently that IL-4 signalling in monocytes and U937 cells involves the activation of a phosphatidylcholine-specific phospholipase C (PC-PLC), but not of phosphatidylinositol 4,5-bisphosphate phospholipase C ($PIP_2$-PLC), $PLA_2$, PLD nor sphingomyelinase.[42] In contrast, our data clearly indicate that IL-4 stimulates an increase in $[Ca^{2+}]_i$ in human promonocytic U937 cells, despite the usual considerable variation which was observed when the response of individual cells was measured. The fluorescent probe we used, Fluo-3 is slightly more convenient for detecting small variations in calcium concentration than Fura-2, used by Ho *et al.*, but this is not sufficient to explain the reasons for this discrepancy. Alternatively there may exist differences between various sub-clones of U937 cells. Our kinetic studies indicate that the late peak of cAMP generation is preceded by an increase in cGMP. At variance with the results observed with rodent monocytes, where a 30 – 100 fold difference between cAMP and cGMP levels has been reported,[43] our data indicate that the basal concentration of cGMP is roughly of the same order of magnitude as that of cGMP, which is more in agreement with the results obtained for human monocytes by Li *et al.*[44] The differential kinetics of the cGMP and cAMP increases suggested a possible link between the accumulation of these two cyclic nucleotides. Indeed, both cGMP and cAMP accumulation were suppressed by L-NMMA and nitro-L-arginine, two inhibitors of the NO synthase (NOS). Nitric oxide (NO), which has been identified in recent years as a pleiotropic mediator,[45–48] is a short-lived radical generated by oxidation of the guanidino group of L-arginine by constitutive or inducible NO synthases.[49–52] Soluble guanylate cyclase is activated by NO through interaction with its haeminic group, which leads to increased cGMP concentration. The subsequent cAMP increase we observed following addition of IL-4 to monocytes could then result from an interplay in the cyclic nucleotide phosphodiesterases (CN-PDE) network. Noteworthy, Li *et al.*[44] have reported that IL-4 and IFN-γ synergistically elicited a marked increase in cAMP phosphodiesterase activity in human monocytes. We have also observed that IL-4 stimulated CN-PDE activity in resting monocytes (not shown). This high CN-PDE activity may help explain the decrease in cAMP and cGMP levels observed in time, despite the presence of IBMX, a nonspecific inhibitor of the various CN-PDE isozymes. Whereas Endres *et al.* observed that CN-PDE inhibitors such as IBMX suppressed the LPS-induced TNF-α production by human mononuclear cells (PBMC) through the accumulation of cAMP,[53] Paul-Eugene *et al.* recently reported that low concentrations of IBMX (10 μM) potentiated the IL-4-driven IgE production and sCD23 release by PBMC.[54]

The cGMP increase might activate the cGMP-inhibitable cAMP-specific phosphodiesterase, (type III CN-PDE), that would result in subsequent cAMP accumulation, independent of the activation of adenylyl cyclase and might explain the delayed phase of cAMP increase. Experiments are in progress to study this mechanism and preliminary results suggest that both chemical NO donators and permeant cGMP analogues, such as Sp-cGMPS, are able to trigger cAMP accumulation in monocytic cells.

Both cGMP and cAMP increases were affected in the presence of EGTA, a calcium chelator, and of W-7 or calmidazolium, two inhibitors of the calcium/calmodulin complex. In contrast with the calcium/calmodulin-dependent activation of constitutive NOS, the activation of inducible forms of NOS (iNOS) is regulated by transcription; once translated, the protein tightly associates with calmodulin, explaining the relative insensitivity of iNOS to modifications of the intracellular calcium concentration.[48] However, the iNOS from human hepatocytes, or Hep-NOS, is partially inhibited by calcium chelators and by antagonists of calmodulin; furthermore, natural and recombinant Hep-NOS might differ in their sensitivity to calcium and calmodulin.[52]

The NO synthase activated by IL-4 in unstimulated monocytes may therefore be of the constitutive type, inasmuch as an endothelial type cNOS mRNA can be detected in these cells by RT-PCR. The presence of both inducible iNOS (type II) and constitutive endothelial NOS (type III) mRNA has been recently reported in human monocytes and monocytic cell lines, such as U937;[55] interestingly, stimulation with IFN-γ and TNF-α led to an induction of iNOS mRNA expression, whereas cNOS mRNA was reduced in the same time. A reciprocal regulation of cNOS and iNOS mRNA has already been observed in other cell types, suggesting the control of their expression by coordinated mechanisms. In our investigation, however, we did not detect a significant reduction of cNOS mRNA following the addition of IFN-γ alone. In addition, treatment with either IL-4, or IL-4 and anti-C23, did not modify the expression of cNOS mRNA in U937 cells. In contrast, these treatments were found to induce the expression of the inducible iNOS, both at the mRNA level, as detected by RT-PCR, and at the protein level, as revealed by immunochemistry (Dugas *et al.*; manuscript in preparation and Reference 56).

Preliminary immunofluorescence experiments, using a mouse monoclonal antibody against anti-human endothelial NOS (Affinity Research Products, Nottingham, UK) confirm the existence of an endothelial type cNOS protein in monocytic cells such as U937. Experiments are in progress to determine if this protein is enzymatically active and whether it is also present in human blood monocytes. Our results also indicate that a calcium increase, by itself, is not sufficient to trigger the activation of cNOS in monocytic cells. They suggest that IL-4 provides other signals or co-factors that are required to obtain a full catalytic activity. One such co-signal could be related to tyrosine phosphorylation, inasmuch as the catalytic activity of another type of constitutive NOS, the neuronal enzyme, is impaired in the presence of tyrosine kinase inhibitors.[57] Indeed, preliminary data suggest that IL-4-induced cGMP accumulation may be impaired by tyrosine kinase inhibitors.

These results indicate that different isoforms of NO synthase, constitutive and inducible, may be expressed in human monocytes, depending on their stage of differentiation and the combination of signals provided.

### References

1. Paul WE, Ohara JB. B cell stimulatory factor-1/interleukin-4. *Ann Rev Immunol* 1987; **5:** 429–459.
2. Te Velde AA, Klomp JPG, Yard BA, De Vries JE, Figdor CG. Modulation of phenotypic and functional properties of human peripheral blood monocytes by IL-4. *J Immunol* 1988; **140:** 1548–1554.
3. Vercelli D, Jabara HH, Le BW, Woodland N, Geha RS, Leung DY. Human recombinant interleukin-4 induces FcεRII/CD23 on normal human monocytes. *J Exp Med* 1988; **167:** 1406–1416.
4. Te Velde AA, Huijbens RJ, De Vries JE, Figdor CG. IL-4 decreases FcγR membrane expression and FcγR-mediated cytotoxicity of human monocytes. *J Immunol* 1990; **144:** 3046–3051.
5. Wong HL, Welch GR, Brandes ME, Wahl SM. IL-4 antagonizes induction of FcγRIII (CD16) expression by transforming growth factor-β on human monocytes. *J Immunol* 1990; **147:** 1843–1848.
6. Lauener RP, Goyert SM, Geha R, Vercelli D. Interleukin-4 down-regulates the expression of CD14 in normal human monocytes. *Eur J Immunol* 1990; **20:** 2375–2379.
7. Te Velde AA, Huijbens RJF, Heije K, De Vries JE, Figdor CG. IL-4 inhibits secretion of IL-1β tumor necrosis factor-α and IL-6 by human monocytes. *Blood* 1990; **76:** 1392–1397.
8. Essner R, Rhoades K, McBride WH, Morton DL, Economou JS. IL-4 down regulates IL-1 and TNF gene expression in human monocytes. *J Immunol* 1989; **142:** 3857–3862.
9. Abramson SL, Gallin JI. IL-4 inhibits superoxide production by human mononuclear phagocytes. *J Immunol* 1990; **144:** 625–630.
10. Lehn M, Weiser WY, Engelborn S, Gillis S, Remhold HG. IL-4 inhibits $H_2O_2$ production and anti-leishmanial capacity of human cultured monocytes mediated by IFN-γ. *J Immunol* 1989; **143:** 3020–3025.
11. Bhaskaran G, Nii A, Sone S, Ogura T. Differential effects of interleukin-4 on superoxide anion production by human alveolar macrophages stimulated with lipopolysaccharide and interferon-γ. *J Leukoc Biol* 1992; **52:** 218–223.
12. Figdor CG, Te Velde AA. Regulation of human phenotype and function by interleukin-4. In: *IL-4: Structure and Function* Spits H, ed. Boca Raton, FL: CRC Press, 1992; 187–199.
13. Wieser M, Bonifer R, Oster W, Lindemann A, Mertelsmann R, Herrmann F. Interleukin-4 induces secretion of CSF for granulocytes and CSF for macrophages by peripheral blood monocytes. *Blood* 1989; **73:** 1105–1109.
14. Idzerda RL, March CJ, Mosley B, *et al.* Human interleukin-4 receptor confers biological responsiveness and defines a novel receptor superfamily. *J Exp Med* 1990; **171:** 861–873.
15. Galizzi JP, Zuber CE, Harada N, *et al.* Molecular cloning of a cDNA encoding the human interleukin-4 receptor. *Int Immunol* 1990; **2:** 669–675.
16. Zurawski SM, Vega Jr F, Huyghe B, Zurawski G. Receptors for interleukin-13 and interleukin-4 are complex and share a novel component that functions in signal transduction. *EMBO J* 1993; **12:** 2663–2670.
17. Aversa G, Punnonen J, Cocks BG, *et al.* An interleukin 4 (IL-4) mutant protein inhibits both IL-4 or IL-13-induced IgE synthesis and B cell proliferation: support for a common component shared by IL-4 and IL-13 receptors. *J Exp Med* 1993; **178:** 2213–2218.
18. Kondo M, Takeshita T, Ishii N, Nakamura M, Watanabe S, Arai K-I, Sugamura K. Sharing of the interleukin-2 (IL-2) receptor γ chain between receptors for IL-2 and IL-4. *Science* 1993; **262:** 1874–1877.
19. Russell SM, Keegan AD, Harada N, *et al.* Interleukin-2 receptor γ chain: a functional component of the interleukin-4 receptor. *Science* 1993; **262:** 1880–1883.
20. Noguchi M, Nakamura Y, Russell SM, Ziegler SF, Tsang M, Cao X, Leonard WJ. Interleukin-2 receptor γ chain: a functional component of the interleukin-7 receptor. *Science* 1993; **262:** 1877–1880.
21. Kawahara A, Minami Y, Taniguchi T. Evidence for a critical role for the cytoplasmic region of the interleukin 2 (IL-2) receptor γ chain in IL-2, IL-4 and IL-7 signalling. *Mol Cell Biol* 1994; **14:** 5433–5440.
22. Watanabe S, Kondo M, Takatsu K, Sugamura K, Arai K-I. Involvement of the interleukin-2 receptor γ subunit in interleukin-4-dependent activation of mouse hematopoietic cells and splenic B cells. *Eur J Immunol* 1995; **25:** 126–131.
23. Wang LM, Keegan AD, Paul WE, Heidaran MA, Gutkind JS, Pierce JH. IL-4 activates a distinct signal transduction cascade from IL-3 in factor-dependent myeloid cells. *EMBO J* 1992; **11:** 4899–4908.
24. Wang LM, Keegan AD, Li W, *et al.* Common elements in interleukin-4 and insulin signalling pathways in factor-dependent hematopoietic cells. *Proc Natl Acad Sci USA* 1993; **90:** 4032–4036.
25. Wang LM, Myers MG Jr, Sun XJ, Aaronson SA, White M, Pierce JH. IRS-1: essential for insulin-and IL-4-stimulated mitogenesis in hematopoietic cells. *Science* 1993; **261:** 1591–1594.
26. Keegan AD, Nelms K, Wang LM, Pierce JH, Paul WE. Interleukin 4 receptor: signaling mechanisms. *Immunology Today* 1994; **15:** 423–431.
27. Keegan AD, Pierce JH. The interleukin-4 receptor: signal transduction by a hematopoietin receptor. *J Leukoc Biol* 1994; **55:** 272–279.
28. Kolb JP, Abadie A. Inhibitors of protein tyrosine kinases and protein tyrosine phosphatases suppress IL-4-induced CD23 expression and release by human B lymphocytes. *Eur Cytokine Netw* 1993; **4:** 429–438.
29. Witthuhn BA, Silvennoinen O, Miura O, *et al.* Involvement of the Jak-3 Janus kinase in signalling by interleukins 2 and 4 in lymphoid and myeloid cells. *Nature* 1991; **370:** 153–157.
30. Musso T, Varesio L, Zhang X, *et al.* IL-4 and IL-13 induce Lsk, a Csk-like tyrosine kinase in human monocytes. *J Exp Med* 1994; **180:** 2383–2388.
31. Kotanides H, Reich NC. Requirement of tyrosine phosphorylation for rapid activation of a DNA binding factor by IL-4. *Science* 1993; **262:** 1265–1267.
32. Schindler C, Kashleva H, Pernis A, Pine R, Rothman P. STF-IL-4: a novel IL-4-induced signal transducing factor. *EMBO J* 1994; **13:** 1350–1356.
33. Kolb JP, Paul-Eugène N, Damais C, Yamaoka K, Drapier JC, Dugas B. Interleukin-4 stimulates cGMP production by IFN-γ-activated human monocytes. Involvement of the nitric oxide synthase pathway. *J Biol Chem* 1994; **269:** 9811–9816.
34. Mautino G, Paul-Eugène N, Chanez P, *et al.* Heterogeneous spontaneous and interleukin-4-induced nitric oxide production by human monocytes. *J Leukoc Biol* 1994; **56:** 15–20.
35. MacDonald RJ, Swith GH, Przbyla AE, Chirgwin JM. Isolation of mRNA using guanidium salts. *Methods Enzymol* 1987; **132:** 407–419.
36. Lasfar A, Amirand C, Ballini JP, Kolb JP. Activation pathways triggered by interleukin-4 in the human plasmacytoma cell line RPMI-8226. Differences with resting B lymphocytes. *Eur Cytokine Netw* 1993; **4:** 213–221.
37. Hart PH, Vitti DR, Burgess GA, Whitty GA, Picolli DS, Hamilton JA. Potential anti-inflammatory effects of IL-4. Suppression of human monocyte tumor necrosis factor, interleukin-1 and prostaglandin $E_2$. *Proc Natl Acad Sci USA* 1989; **63:** 3808–3812.
38. de Wit H, Hendriks DW, Halie MR, Vellenga E. Interleukin-4 receptor regulation in human monocytic cells. *Blood* 1994; **84:** 608–615.
39. Takeshita T, Asao H, Ohtani N, *et al.* Cloning of the γ chain of the human IL-2 receptor. *Science* 1992; **257:** 379–382.
40. Rasmussen H, Isales C, Ganesan S, Calle R, Zawalich W. $Ca^{2+}$–cyclic AMP interactions in sustained cellular responses. In: *Interactions Among Cell Signalling Systems.* Ciba Foundation Symposium **164**. Chichester: John Wiley and Sons, 1992; 98–112.
41. Arruda S, Ho JL. IL-4 receptor signal transduction in human monocytes is associated with protein kinase C translocation. *J Immunol* 1992; **149:** 1258–1264.
42. Ho JL, Zhu B, He S, Du B, Rothman R. Interleukin 4 receptor signaling in human monocytes and U937 cells involves the activation of a phosphatidylcholine specific phospholipase C: a comparison with chemotactic peptide, FMLP, phospholipase D, and sphingomyelinase. *J Exp Med* 1994; **180:** 1457–1469.
43. Renz H, Gong J-H, Schmidt A, Nain M, Gemsa D. Release of tumor necrosis factor-α from macrophages. Enhancement and suppression are dose-dependently regulated by prostaglandin $E_2$ and cyclic nucleotides. *J Immunol* 1988; **141:** 2388-2393.
44. Li S-H, Chan SC, Toshitani A, Leung DYM, Hanifin JM. Synergistic effects of interleukin 4 and interferon-gamma on monocyte phosphodiesterase activity. *J Invest Dermatol* 1992; **99:** 65–70.
45. Moncada S, Palmer RMJ, Higgs EA. Nitric oxide: physiology, pathophysiology and pharmacology. *Pharmacol Rev* 1991; **43:** 109–144.
46. Nathan CF. Nitric oxide as secretory product of mammalian cells. *FASEB J* 1992; **6:** 3051–3064.
47. Nathan CF, Hibbs JB Jr. Role of nitric oxide synthesis in macrophage antimicrobial activity. *Current Opin in Immunol* 1991; **3:** 65–70.
48. Stuehr DJ, Griffith OW. Mammalian NO synthases. *Adv Enzymol* 1992; **65:** 287–346.
49. Lowenstein C, Glatt CS, Bredt DS, Snyder SH. Cloned and expressed macrophage nitric oxide synthase contrasts with the brain enzyme. *Proc Natl Acad Sci USA* 1992; **89:** 6711–6715.
50. Lyons CR, Ofloff GJ, Cunningham JM. Molecular cloning and functional expression of an inducible nitric oxide synthase from a murine macrophage cell line. *J Biol Chem* 1992; **267:** 6370–6374.
51. Xie QW, Cho HJ, Calaycay J, *et al.* Cloning and characterization of inducible nitric oxide synthase from mouse macrophages. *Science* 1992; **256:** 225–228.
52. Geller DA, Lowenstein CJ, Shapiro RA, *et al.* Molecular cloning and expression of inducible nitric oxide synthase from human hepatocytes. *Proc Natl Acad Sci USA* 1993; **90:** 3491–3495.
53. Endres S, Fülle H-J, Sinha D, *et al.* Cyclic nucleotides differentially regulate the synthesis of tumour necrosis factor-α and interleukin-1β by human mononuclear cells. *Immunology* 1991; **72:** 56–60.
54. Paul-Eugène N, Pène J, Bousquet J, Dugas B. Role of cyclic nucleotides and nitric oxide in blood mononuclear cell IgE production stimulated by IL-4. *Cytokine* 1995; **7:** 64–69.
55. Reiling N, Ulmer AJ, Duchrow M, Ernst M, Flad HD, Hauschildt S. Nitric oxide synthase: mRNA expression of different isoforms in human monocytes/macrophages. *Eur J Immunol* 1994; **24:** 1941–1944.
56. Vouldoukis I, Riveros-Moreno V, Dugas B, *et al.* The killing of *Leishmania major* by human macrophages is mediated by nitric oxide induced after ligation of FcεRII/CD23 surface antigen. *Proc Natl Acad Sci USA* 1995. In press.
57. Rodriguez J, Quignard JF, Fagni L, Lafon-Cazal M, Bockaert J. Blockade of nitric oxide synthesis by tyrosine kinase inhibitors in neurones. *Neuropharmacology* 1994; **33:** 1267–1274.

ACKNOWLEDGMENTS. This work was supported by INSERM and by a grant from ARC.

**Received 9 March 1995;
accepted in revised form 11 May 1995**

## References

[PDF_00835] Arruda S., Ho J. L. (1992). IL-4 receptor signal transduction in human monocytes is associated with protein kinase C translocation.. J Immunol.

[PDF_00765] Aversa G., Punnonen J., Cocks B. G., de Waal Malefyt R., Vega F., Zurawski S. M., Zurawski G., de Vries J. E. (1993). An interleukin 4 (IL-4) mutant protein inhibits both IL-4 or IL-13-induced human immunoglobulin G4 (IgG4) and IgE synthesis and B cell proliferation: support for a common component shared by IL-4 and IL-13 receptors.. J Exp Med.

[PDF_00745] Bhaskaran G., Nii A., Sone S., Ogura T. (1992). Differential effects of interleukin-4 on superoxide anion production by human alveolar macrophages stimulated with lipopolysaccharide and interferon-gamma.. J Leukoc Biol.

[PDF_00870] Endres S., Fülle H. J., Sinha B., Stoll D., Dinarello C. A., Gerzer R., Weber P. C. (1991). Cyclic nucleotides differentially regulate the synthesis of tumour necrosis factor-alpha and interleukin-1 beta by human mononuclear cells.. Immunology.

[PDF_00737] Essner R., Rhoades K., McBride W. H., Morton D. L., Economou J. S. (1989). IL-4 down-regulates IL-1 and TNF gene expression in human monocytes.. J Immunol.

[PDF_00759] Galizzi J. P., Zuber C. E., Harada N., Gorman D. M., Djossou O., Kastelein R., Banchereau J., Howard M., Miyajima A. (1990). Molecular cloning of a cDNA encoding the human interleukin 4 receptor.. Int Immunol.

[PDF_00867] Geller D. A., Lowenstein C. J., Shapiro R. A., Nussler A. K., Di Silvio M., Wang S. C., Nakayama D. K., Simmons R. L., Snyder S. H., Billiar T. R. (1993). Molecular cloning and expression of inducible nitric oxide synthase from human hepatocytes.. Proc Natl Acad Sci U S A.

[PDF_00838] Ho J. L., Zhu B., He S., Du B., Rothman R. (1994). Interleukin 4 receptor signaling in human monocytes and U937 cells involves the activation of a phosphatidylcholine-specific phospholipase C: a comparison with chemotactic peptide, FMLP, phospholipase D, and sphingomyelinase.. J Exp Med.

[PDF_00756] Idzerda R. L., March C. J., Mosley B., Lyman S. D., Vanden Bos T., Gimpel S. D., Din W. S., Grabstein K. H., Widmer M. B., Park L. S. (1990). Human interleukin 4 receptor confers biological responsiveness and defines a novel receptor superfamily.. J Exp Med.

[PDF_00794] Keegan A. D., Nelms K., Wang L. M., Pierce J. H., Paul W. E. (1994). Interleukin 4 receptor: signaling mechanisms.. Immunol Today.

[PDF_00796] Kolb J. P., Abadie A. (1993). Inhibitors of protein tyrosine kinases and protein tyrosine phosphatases suppress IL-4-induced CD23 expression and release by human B lymphocytes.. Eur Cytokine Netw.

[PDF_00811] Kolb J. P., Paul-Eugene N., Damais C., Yamaoka K., Drapier J. C., Dugas B. (1994). Interleukin-4 stimulates cGMP production by IFN-gamma-activated human monocytes. Involvement of the nitric oxide synthase pathway.. J Biol Chem.

[PDF_00769] Kondo M., Takeshita T., Ishii N., Nakamura M., Watanabe S., Arai K., Sugamura K. (1993). Sharing of the interleukin-2 (IL-2) receptor gamma chain between receptors for IL-2 and IL-4.. Science.

[PDF_00806] Kotanides H., Reich N. C. (1993). Requirement of tyrosine phosphorylation for rapid activation of a DNA binding factor by IL-4.. Science.

[PDF_00818] Lasfar A., Amirand C., Ballini J. P., Kolb J. P. (1993). Activation pathways triggered by interleukin-4 in the human plasmacytoma cell line RPMI-8226--differences with resting B lymphocytes.. Eur Cytokine Netw.

[PDF_00731] Lauener R. P., Goyert S. M., Geha R. S., Vercelli D. (1990). Interleukin 4 down-regulates the expression of CD14 in normal human monocytes.. Eur J Immunol.

[PDF_00858] Lowenstein C. J., Glatt C. S., Bredt D. S., Snyder S. H. (1992). Cloned and expressed macrophage nitric oxide synthase contrasts with the brain enzyme.. Proc Natl Acad Sci U S A.

[PDF_00861] Lyons C. R., Orloff G. J., Cunningham J. M. (1992). Molecular cloning and functional expression of an inducible nitric oxide synthase from a murine macrophage cell line.. J Biol Chem.

[PDF_00847] Moncada S., Palmer R. M., Higgs E. A. (1991). Nitric oxide: physiology, pathophysiology, and pharmacology.. Pharmacol Rev.

[PDF_00804] Musso T., Varesio L., Zhang X., Rowe T. K., Ferrara P., Ortaldo J. R., O'Shea J. J., McVicar D. W. (1994). IL-4 and IL-13 induce Lsk, a Csk-like tyrosine kinase, in human monocytes.. J Exp Med.

[PDF_00852] Nathan C. (1992). Nitric oxide as a secretory product of mammalian cells.. FASEB J.

[PDF_00775] Noguchi M., Nakamura Y., Russell S. M., Ziegler S. F., Tsang M., Cao X., Leonard W. J. (1993). Interleukin-2 receptor gamma chain: a functional component of the interleukin-7 receptor.. Science.

[PDF_00873] Paul-Eugène N., Pène J., Bousquet J., Dugas B. (1995). Role of cyclic nucleotides and nitric oxide in blood mononuclear cell IgE production stimulated by IL-4.. Cytokine.

[PDF_00715] Paul W. E., Ohara J. (1987). B-cell stimulatory factor-1/interleukin 4.. Annu Rev Immunol.

[PDF_00831] Rasmussen H., Isales C., Ganesan S., Calle R., Zawalich W. (1992). Ca(2+)-cyclic AMP interactions in sustained cellular responses.. Ciba Found Symp.

[PDF_00876] Reiling N., Ulmer A. J., Duchrow M., Ernst M., Flad H. D., Hauschildt S. (1994). Nitric oxide synthase: mRNA expression of different isoforms in human monocytes/macrophages.. Eur J Immunol.

[PDF_00883] Rodriguez J., Quignard J. F., Fagni L., Lafon-Cazal M., Bockaert J. (1994). Blockade of nitric oxide synthesis by tyrosine kinase inhibitors in neurones.. Neuropharmacology.

[PDF_00772] Russell S. M., Keegan A. D., Harada N., Nakamura Y., Noguchi M., Leland P., Friedmann M. C., Miyajima A., Puri R. K., Paul W. E. (1993). Interleukin-2 receptor gamma chain: a functional component of the interleukin-4 receptor.. Science.

[PDF_00809] Schindler C., Kashleva H., Pernis A., Pine R., Rothman P. (1994). STF-IL-4: a novel IL-4-induced signal transducing factor.. EMBO J.

[PDF_00854] Stuehr D. J., Griffith O. W. (1992). Mammalian nitric oxide synthases.. Adv Enzymol Relat Areas Mol Biol.

[PDF_00829] Takeshita T., Asao H., Ohtani K., Ishii N., Kumaki S., Tanaka N., Munakata H., Nakamura M., Sugamura K. (1992). Cloning of the gamma chain of the human IL-2 receptor.. Science.

[PDF_00720] Vercelli D., Jabara H. H., Lee B. W., Woodland N., Geha R. S., Leung D. Y. (1988). Human recombinant interleukin 4 induces Fc epsilon R2/CD23 on normal human monocytes.. J Exp Med.

[PDF_00788] Wang L. M., Keegan A. D., Li W., Lienhard G. E., Pacini S., Gutkind J. S., Myers M. G., Sun X. J., White M. F., Aaronson S. A. (1993). Common elements in interleukin 4 and insulin signaling pathways in factor-dependent hematopoietic cells.. Proc Natl Acad Sci U S A.

[PDF_00785] Wang L. M., Keegan A. D., Paul W. E., Heidaran M. A., Gutkind J. S., Pierce J. H. (1992). IL-4 activates a distinct signal transduction cascade from IL-3 in factor-dependent myeloid cells.. EMBO J.

[PDF_00791] Wang L. M., Myers M. G., Sun X. J., Aaronson S. A., White M., Pierce J. H. (1993). IRS-1: essential for insulin- and IL-4-stimulated mitogenesis in hematopoietic cells.. Science.

[PDF_00781] Watanabe S., Kondo M., Takatsu K., Sugamura K., Arai K. (1995). Involvement of the interleukin-2 receptor gamma subunit in interleukin-4-dependent activation of mouse hematopoietic cells and splenic B cells.. Eur J Immunol.

[PDF_00752] Wieser M., Bonifer R., Oster W., Lindemann A., Mertelsmann R., Herrmann F. (1989). Interleukin-4 induces secretion of CSF for granulocytes and CSF for macrophages by peripheral blood monocytes.. Blood.

[PDF_00728] Wong H. L., Welch G. R., Brandes M. E., Wahl S. M. (1991). IL-4 antagonizes induction of Fc gamma RIII (CD16) expression by transforming growth factor-beta on human monocytes.. J Immunol.

[PDF_00864] Xie Q. W., Cho H. J., Calaycay J., Mumford R. A., Swiderek K. M., Lee T. D., Ding A., Troso T., Nathan C. (1992). Cloning and characterization of inducible nitric oxide synthase from mouse macrophages.. Science.

[PDF_00762] Zurawski S. M., Vega F., Huyghe B., Zurawski G. (1993). Receptors for interleukin-13 and interleukin-4 are complex and share a novel component that functions in signal transduction.. EMBO J.

[PDF_00827] de Wit H., Hendriks D. W., Halie M. R., Vellenga E. (1994). Interleukin-4 receptor regulation in human monocytic cells.. Blood.

[PDF_00734] te Velde A. A., Huijbens R. J., Heije K., de Vries J. E., Figdor C. G. (1990). Interleukin-4 (IL-4) inhibits secretion of IL-1 beta, tumor necrosis factor alpha, and IL-6 by human monocytes.. Blood.

[PDF_00725] te Velde A. A., Huijbens R. J., de Vries J. E., Figdor C. G. (1990). IL-4 decreases Fc gamma R membrane expression and Fc gamma R-mediated cytotoxic activity of human monocytes.. J Immunol.

[PDF_00717] te Velde A. A., Klomp J. P., Yard B. A., de Vries J. E., Figdor C. G. (1988). Modulation of phenotypic and functional properties of human peripheral blood monocytes by IL-4.. J Immunol.

